# An algorithm for surgical treatment of children with bone sarcomas of the extremities

**DOI:** 10.1051/sicotj/2024033

**Published:** 2024-10-04

**Authors:** Costantino Errani, Ahmed Atherley O’Meally, Shinji Tsukamoto, Andreas F. Mavrogenis, Yasuhito Tanaka, Marco Manfrini

**Affiliations:** 1 Department of Orthopaedic Oncology, IRCCS Istituto Ortopedico Rizzoli Via Pupilli 1 Bologna 40136 Italy; 2 Department of Orthopaedic Surgery, Nara Medical University 840, Shijo-cho, Kashihara Nara 634-8521 Japan; 3 First Department of Orthopaedics, National and Kapodistrian University of Athens, School of Medicine 41 Ventouri Street, Holargos Athens 15562 Greece

**Keywords:** Bone sarcoma, Limb salvage surgery, Children, Massive bone allograft, Vascularized fibula, Prosthetic reconstruction

## Abstract

*Introduction*: Limb salvage surgery in children following bone sarcoma resection is a challenging problem because of the small size of the bones, the lack of appropriate size-matched implants, and the risk of limb-length discrepancy once skeletal growth is complete, secondary to the loss of the epiphyseal plate. Although several reconstruction options are available in children with bone sarcomas, such as vascularized fibula, massive bone allograft, extracorporeal devitalized autograft, endoprosthesis, and allograft-prosthesis composite, a consensus has not been reached on the best reconstruction method. The purpose of the present study is to propose an algorithm for reconstruction after resection of bone sarcomas in children. *Methods*: In this review, we analyzed reports on limb reconstruction in children following treatment for bone sarcoma, to provide a comprehensive overview of the different reconstruction options in children with bone sarcomas, the outcomes, and the risks and benefits of the different surgical approaches. *Results*: Despite a high risk of complications and the necessity for limb-lengthening procedures, prosthetic or biological reconstructions seem to achieve good functional outcomes in children with bone sarcoma. The use of massive bone graft seems to be recommended for intercalary reconstructions, with a free vascularized fibular graft for long defects, while for osteoarticular reconstruction a modular or expandable prosthesis or an allograft–prosthesis composite seems to have good results. For reconstruction of the proximal humerus, modular prosthesis or allograft-prosthesis composite are more commonly used than expandable prosthesis since there are fewer functional constraints related to possible limb-length discrepancy on the upper limb compared to the lower limb. *Discussion*: We discuss the advantages and limitations of the different available surgical options for bone reconstruction following tumor resection in children and propose an algorithm of potential surgical treatments for children with bone sarcomas of the extremities.

## Introduction

Limb salvage surgery in children after resection of bone sarcoma is a challenge for surgeons due to small bone size, lack of size-matched implants, and possible limb-length discrepancy when the patient reaches skeletal maturity secondary to loss of the growth plate [1, 2]. Reconstruction options following bone sarcoma resection in children include biological reconstructions (such as free vascularized fibular graft [FVFG], massive bone allograft [MBA], and tumor-devitalized autograft) and prosthetic reconstructions (such as a modular or expandable prosthesis or an allograft–prosthesis composite) [3, 4]. At present there is no consensus concerning the best reconstruction option in children with bone sarcomas of the extremities [5]. Biological reconstruction with an MBA, tumor-devitalized autograft and FVFG is generally regarded as the gold standard following intercalary resection of bone sarcomas in children [3, 6–8], while prosthetic reconstruction with a modular or expandable prosthesis or an allograft–prosthetic composite seems to be considered the gold standard following osteoarticular resection [9–12]. Here, we provide an overview of the different reconstruction options, discuss their advantages and limitations, and propose an algorithm for surgical treatments of children with bone sarcoma of the extremities.

## Intercalary reconstructions

**Figure 1:**
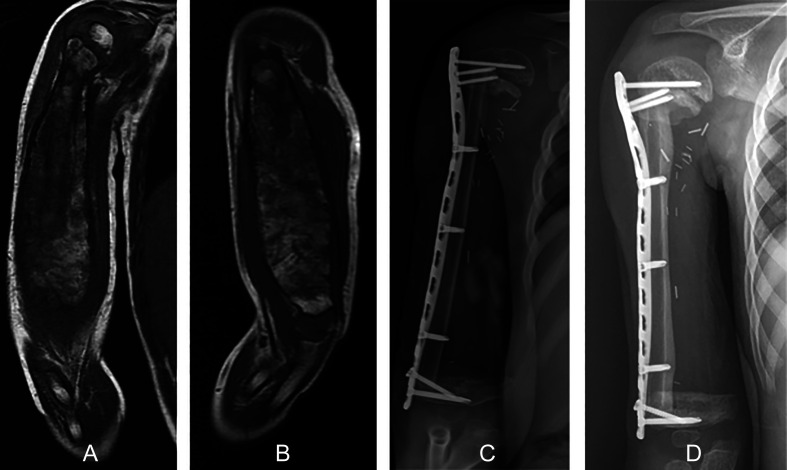
A six-year-old boy, diagnosed with Ewing sarcoma, who was treated by intercalary resection followed by reconstruction of the right humerus using a free-vascularized fibular graft. Coronal (A) and sagittal (B) T1 magnetic resonance images show a tumor located at the diaphyseal region of the humerus without involvement of the proximal or distal growth plates. A postoperative anteroposterior radiographic view (C) shows an intercalary reconstruction of the humerus with a free-vascularized fibular graft. (D) Radiograph showing initial consolidation of the fibular graft at the two-month follow-up. Although fixation was possible with just a few screws, the fibula graft was able to integrate within two months, making the reconstruction stable.

Reconstruction options in children following intercalary resection of bone sarcomas include biologic reconstructions (such as distraction osteogenesis, tumor-devitalized autograft or MBA with or without an FVFG) or intercalary modular prosthetic reconstructions [3, 7, 13–20]; in general, biologic reconstructions are preferred (Fig. 1) [3, 17, 21, 22] because they allow preservation of the bone stock [13]. Although distraction osteogenesis is an alternative biological reconstruction option, it is time-consuming and requires additional surgeries, making it less suitable for children with large bone defects undergoing chemotherapy [19].

A tumor-devitalized autograft represents a useful reconstruction option, especially in low-income countries that have limited access to bone donations and lack bone banks [23]. Several techniques are used to produce tumor-devitalized autografts such as extra-corporeal radiation, autoclaving, pasteurization, and deep freezing [23]. Among these, frozen autografts appear superior to autoclaved or pasteurized autografts [24]. Histological analysis of autoclaved autografts, performed two years post-surgery, showed that most were not incorporated [25], analysis of frozen autografts more than one year post-surgery, found osteocytes and osteoblasts infiltrated into a wide area of the graft and bone formation at the host bone–graft junction [24]. The main advantage of tumor-devitalized autografts is that the size of the autograft perfectly matches the host bone [23], the main disadvantage lies in the inability to analyze surgical margins and evaluate the patient’s response to chemotherapy [18]. Furthermore, in some patients with severe cortical bone destruction, bone recycling is not possible [18]. The complication risk and graft survival are similar between tumor-devitalized autografts and MBAs, including non-union, infection, fracture, and bone resorption [23, 26, 27].

**Figure 2:**
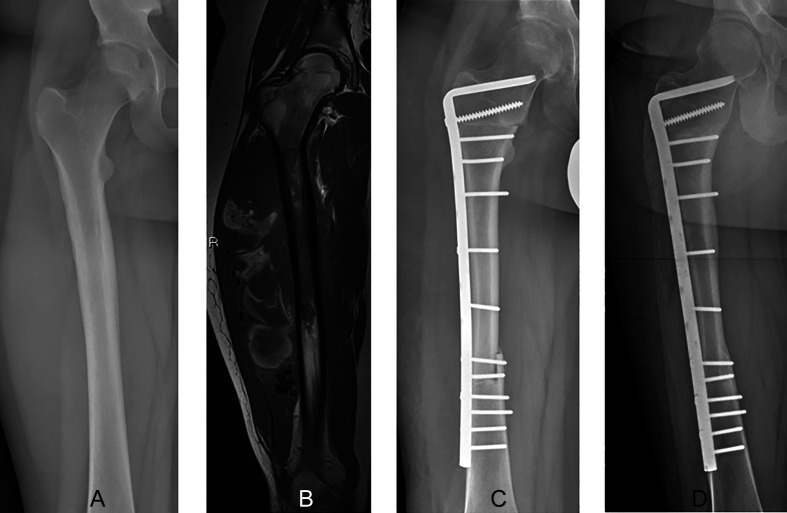
A 16-year-old boy with a diagnosis of Ewing sarcoma of the right femur. Preoperative anteroposterior radiographic view (A) showing a lytic lesion with periosteal reaction at the proximal diaphyseal region of the right femur. Coronal-T1 (B) magnetic resonance image shows tumor extension and a large extraosseous mass at the proximal diaphysis without involvement of the meta-epiphyseal region of the femur. Radiographs showing intercalary reconstruction of the femur using a massive bone allograft at the three-month (C) and 8-year (D) follow-up, showing consolidation and integration of the massive bone allograft. The massive bone allograft was able to be incorporated into the host bone despite the absence of the vascularized fibula graft as the resection was less than 15 cm.

An MBA seems to be the preferred biological reconstruction method following intercalary resection in children (Fig. 2) [3, 28]. The main advantage of this method is the preservation of bone stock [18, 29]. Previous studies showed that MBAs can be incorporated into host bone, surviving for decades, most likely because the external surface is gradually populated with live cells and therefore becomes “revascularized”. An MBA may act as a scaffold allowing revascularization from both ends of the graft, however, this usually needs a very long time [7, 30–32]. The use of MBAs may have a high risk of complications including nonunion, fracture, and infection [3, 7, 17]. Because MBAs are acellular and lack a blood supply, spontaneous healing following fracture or nonunion is not possible [33], and intercalary MBA reconstruction in the femur seems to be particularly at a higher risk of failure than other long bones [13, 34, 35]. Intercalary reconstruction in the tibia may be possible using an MBA in combination with ipsilateral pedicled vascularized fibular transfer instead of an FVFG, reducing the operation time [3, 15]. Previous studies analyzing intercalary reconstruction of the femur showed that the combination of an MBA with an FVFG may be associated with longer operative times (due to graft harvest) and infection risk, as well as morbidity at the donor site and stress fractures of the vascularized fibula [3, 36]. Additionally, harvest of the fibular graft has been associated with valgus-ankle deformity or flexion deformity of the big toe, which occurred in up to 17% of patients [29, 36].

**Figure 3:**
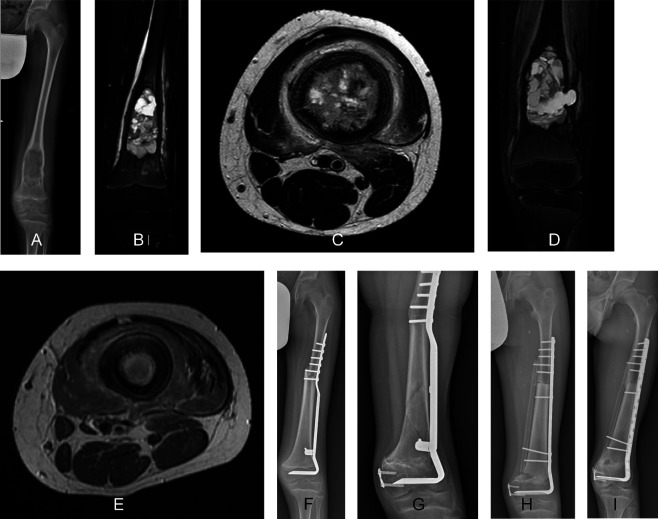
A 12-year-old boy, diagnosed with osteosarcoma of the left femur, was treated with intercalary resection and massive bone allograft reconstruction. (A) An anteroposterior radiographic view at presentation showing a lytic lesion located at the distal diaphyseal region of the femur, without involvement of the physis. The edema around the lesion seen in the anteroposterior T2 Short-TI Inversion Recovery magnetic resonance image (B) and the axial T2 magnetic resonance image (C) before preoperative chemotherapy disappeared in the anteroposterior T2 Short-TI Inversion Recovery magnetic resonance image (D) and the axial T2 magnetic resonance image (E) after preoperative chemotherapy. (F) Radiograph showing consolidation of the intercalary allograft at the 3-month follow-up. Radiograph showing allograft fracture 2 years after the surgery (G) treated using a new massive bone allograft in combination with a free vascularized fibular graft (H). (I) Radiographic view showing consolidation and integration of the allograft with the fibular graft 2 years after the surgery. The vascularized fibula graft could serve as an alternative for addressing fracture of intercalary massive bone allograft.

Some studies reported that nonunion may be more frequent following intercalary MBA reconstruction alone compared with intercalary MBA combined with FVFG reconstruction [3, 7, 17]. An FVFG may help bone healing and union by promoting bone remodeling and fibular graft hypertrophy [33]. Houdek et al. found no difference in the occurrence of nonunion between children treated with intercalary MBA reconstruction alone (36% of children) and those treated with intercalary MBA with FVFG reconstruction (33% of children) [32]. Another retrospective study confirmed no difference in nonunion rates between the two reconstruction options [4], but an FVFG was added to the MBA reconstruction for defects longer than 15 cm [4]. Therefore, the authors suggested that an FVFG could serve as an alternative for addressing fractures or nonunion in intercalary MBA reconstruction alone (Fig. 3) [4].

Fractures of a massive bone autograft or allograft may occur in as many as 20% of patients after intercalary reconstruction following resection of bone sarcomas [3, 7, 36]. A retrospective study of 83 patients treated with intercalary allograft reconstruction in the femur reported fractures in 17% of patients, resulting in the removal of the reconstruction in all but one patient [7]. Allograft fracture shows some correlation to the patient’s age (>18 years) and length of bone resected (>17 cm) [35]. Several studies reported no difference regarding the risk of graft fracture in patients who received an intercalary MBA reconstruction with or without an FVFG reconstruction [3, 13, 17, 32, 36, 37]. In a case series of 29 children with bone sarcomas of the lower extremities who underwent intercalary resection, there was a similar fracture risk in children treated with an MBA between those who received no other graft and those who also received a vascularized fibular graft, at 44% and 45% respectively [32]. A retrospective study examined children who underwent intercalary reconstruction of the femur after resection of a bone tumor [4]. Among the children treated with intercalary allograft reconstruction alone, graft fractures occurred in five of 21 (24%) children, while graft fractures occurred in seven of 25 (28%) children with intercalary bone allograft and FVFG reconstruction (OR 0.8 [95% CI 0.2 to 3.0]; *p* > 0.99), showing no difference in the fracture risk [4]. This study suggested that an FVFG may help preserve the reconstruction in most children who had a fracture when initially reconstructed with an intercalary bone allograft and free FVFG. By contrast, in most children reconstructed with an intercalary bone allograft alone, revision of the reconstruction was necessary following graft fracture [4, 32].

Infection has been reported in up to 18% of patients following intercalary MBA reconstruction [7, 30, 34]. Prolonged operative times due to harvesting of the free fibular graft and vascular anastomosis seem to increase the risk of infection in patients reconstructed using an intercalary MBA together with an FVFG [29]. However, other studies reported that the risk of infection in these two treatment groups was similar [3, 29, 38, 39]. In another study, no infection was reported in children treated with an MBA and an FVFG compared to a 7% infection risk in those who received an MBA alone [32], while in contrast, a retrospective study of children with intercalary femur reconstruction reported a higher risk of infection among children treated with an intercalary bone allograft and an FVFG compared to those who did not receive an FVFG. However, none of those with the FVFG needed reconstruction removal [4].

## Osteoarticular reconstructions

Reconstruction options for children following osteoarticular resection of bone sarcomas include osteoarticular allograft, modular or expandable prosthesis, and allograft–prosthesis composite [2, 5, 9–12, 22, 40]. Osteoarticular allograft reconstruction is ideal for the preservation of the bone stock and possible soft tissue reattachment site [2, 22]. However, it is associated with the risk of complications such as poor cartilage viability, nonunion, infection, and fracture [2, 22, 41]. Additionally, size-matching of osteoarticular allografts in children is an important limitation [21]. The use of a modular or expandable prosthesis requires less technically demanding surgery and a shorter rehabilitation period [5, 9, 12]. A modular prosthesis or expandable prosthesis may be utilized when the dimensions of the prosthesis fit the size of the host bone in children [21]. However, in very young children, where the bone is small and a prosthetic reconstruction is not possible, allograft–prosthesis composite reconstruction may be considered [10]. This reconstruction combines the advantages of prosthetic and biological reconstructions, providing a good soft-tissue attachment site, and avoiding problems caused by the size of an MBA, while also preserving the bone stock for future revision surgeries [10, 11, 21]. Complications reported with these reconstruction options include infection, dislocation, aseptic loosening, fracture, nonunion, and resorption [5, 9, 40]. Another challenge during the reconstruction of very young and still-growing children lies in the risk of limb-length discrepancy at the end of growth as a result of sacrifice of the physis in patients who are skeletally immature [42, 43]. There have been a few studies analyzing children with bone sarcomas of the extremities treated with prosthetic or allograft–prosthesis composite reconstruction following osteoarticular resections, showing that survival and functional outcomes after both reconstruction options are similar [10–12, 44].

## Knee

**Figure 4:**
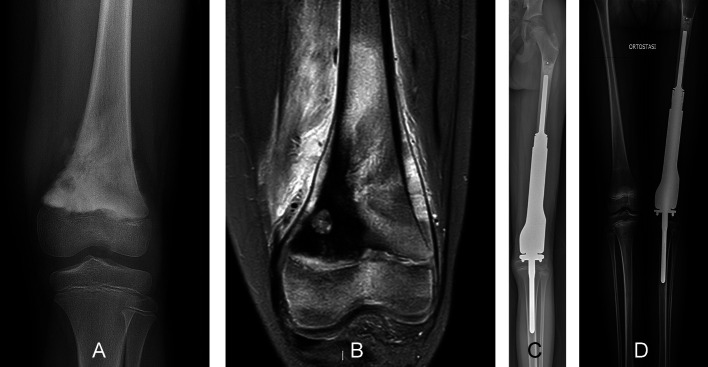
A 10-year-old boy, diagnosed with osteosarcoma of the left distal femur, who was treated by osteoarticular resection followed by reconstruction using an expandable modular prosthesis. (A) An anteroposterior radiographic view showed osteosarcoma of the distal femur infiltrating the growth plate. (B) Coronal-fat saturation magnetic resonance image showing the location of the tumor at the distal femur. (C) Postoperative radiographs showing reconstruction of the distal femur with an expandable modular prosthesis at the 5-month follow-up. (D) Radiograph of the lower limbs showing elongation of the modular prosthesis at the 2-year follow-up. The extendable prosthesis was able to avoid discrepancies in children undergoing resection of the distal femur with sacrifice of the growth plate.

**Figure 5:**
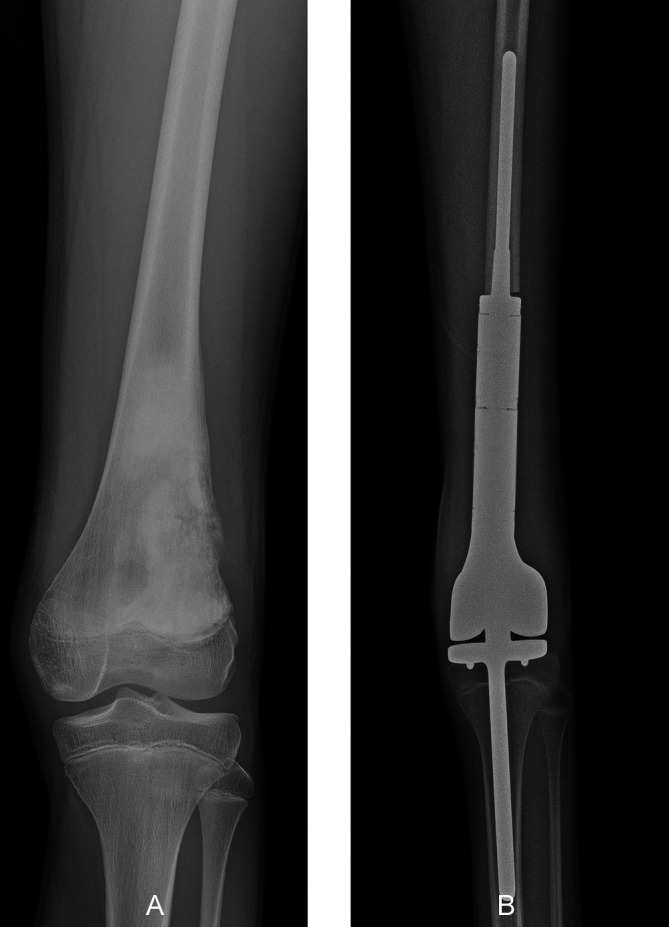
A 12-year-old boy diagnosed with osteosarcoma of the left distal femur, who was treated by osteoarticular resection followed by modular prosthesis reconstruction. (A) Preoperative anteroposterior radiographic view of the knee showing a permeating sclerotic lesion of the distal femur. (B) An immediate postoperative radiograph showing reconstruction of the distal femur using a modular prosthesis with a smooth tibial stem. The modular distal femur prosthesis with smooth tibial stem was able to preserve the growth of the growth plate of the proximal tibia in children with sarcoma of the distal femur.

The growth plates at the distal femur and proximal tibia contribute more than 67% of the longitudinal growth of the lower limb [11]; therefore, tumor resection in this region significantly impacts leg growth and ultimate length at skeletal maturity [45]. In very young children (<5 years), rotationplasty is still a surgical option, and expandable prostheses have been designed to overcome resultant limb length discrepancy in children aged between 6 and 9 (Fig. 4) [12, 43, 46]. However, recent studies reported a high risk of complications and bone loss with these prostheses [44, 47, 48]. A retrospective study of ten children treated by resection of the distal femur followed by reconstruction using an expandable prosthesis reported 37 complications requiring 15 reoperations [44]. Reconstruction failure occurred in six children, among whom four were treated with a modular prosthesis and two with a total femoral prosthesis. Aseptic loosening was the predominant mode of failure [44]. During the prosthetic reconstruction of the distal femur, fixation of the prosthetic reconstruction in the ipsilateral proximal tibia requires that a portion of the stem of the prosthesis crosses the physis, potentially slowing or completely stopping its growth [47–49]. Therefore, using a smooth press-fit stem may help preserve the growth of the proximal tibial physis following tumor resection of the distal femur (Fig. 5) [47]. A retrospective study reported the outcomes of 23 children who received distal femoral reconstruction with an expandable prosthesis using a cemented or uncemented smooth tibial stem [48]. All children exhibited some growth at the proximal tibial physis after surgery, but 65% of children exhibited shortening of the tibial length with a median reduction of 9.1 mm, representing a reduction of 2.5 mm per year of growth [48]. By contrast, another retrospective study analyzing six children treated with a distal femur expandable prosthesis, showed that implantation of a press-fit smooth stem through the uninvolved adjacent physis did not affect physical growth [47].

Among 94 children who underwent reconstruction with a cementless modular prosthesis (62 distal femur and 32 proximal tibia) the 8-year cumulative incidence of revision surgery for any cause was 32% (95% CI [23% to 42%]). The 8-year cumulative incidence of complications was: aseptic loosening, 5% (95% CI [2% to 11%]); periprosthetic fracture, 9% (95% CI [4% to 15%]); hinge breakage, 19% (95% CI [12% to 28%]), and infection, 7% (95% CI [3% to 14%]) [49]. The authors reported that 26% of children had cortical atrophy around the implanted stems, potentially leading to complications such as loosening or fracture in the future [49]. Cortical atrophy is another common problem after prosthetic reconstruction to treat bone sarcomas [50]. A retrospective study reviewed 31 stems in 19 children treated with resection and modular prosthesis reconstruction and found cortical atrophy in 13 stems within 3 years. A stem–cortex diameter ratio of ≥0.5 and patient age below 10 years was associated with the occurrence of cortical atrophy (with p values of 0.002 and 0.019, respectively), associated with an increased risk of reconstruction failure: nine of 13 children in the cortical atrophy group compared to four of 18 children in the non-cortical atrophy group (*p* = 0.035) [50].

**Figure 6:**
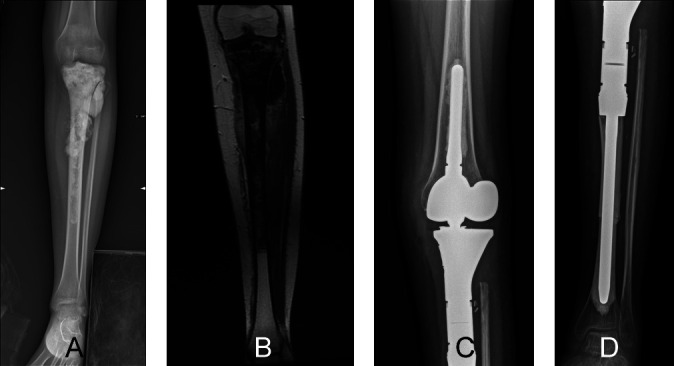
A 17-year-old girl diagnosed with osteosarcoma of the left proximal tibia who was treated by osteoarticular resection and modular prosthesis reconstruction. (A) Preoperative anteroposterior radiographic view showing a permeating sclerotic lesion of the proximal tibia. (B) A coronal-T1 magnetic resonance image showing the tumor located at the proximal tibia that extends to the diaphysis. Postoperative radiographs (C and D) show modular prosthesis reconstruction and intercalary massive bone allograft augmentation of the proximal tibia at the 12-month follow-up. The modular prosthesis was one of the treatments of choice in children at the end of their skeletal growth undergoing joint resection for bone sarcoma.

Modular prosthesis, MBA, and allograft–prosthesis composite are the reconstruction options following the resection of the proximal tibia (Fig. 6) [51]. A modular prosthesis requires the removal not only of the physis of the proximal tibia but also of the distal femur, leading to dysmetria of the lower limbs once the skeleton has stopped growing [51, 52]. In addition, a modular prosthesis may carry a high risk of cortical atrophy and loosening, possibly necessitating several revision surgeries [52]. An osteoarticular allograft has a high failure risk and the problem of mismatch with the host bone [51, 52]. The most frequent complications with osteoarticular allografts are subchondral and metaphyseal graft fractures, reported in up to 80% of patients [52, 53]. In contrast an allograft–prosthesis composite spares the distal femoral physis and allows the MBA to match the size of the tibia in children with bone sarcomas [51, 52]. Other potential benefits of an allograft–prosthesis composite include restoration of the bone stock, the possibility of reattaching tendons to the graft, as well as improved longevity due to the graft’s load-sharing properties [41]. In 19 children treated with an allograft–prosthesis composite, 13 retained the original reconstruction, and most achieved good functional outcomes. The risk of allograft fracture was 32% and the limb length discrepancy was 1.9 cm (ranging from 0.5 to 4 cm) [52].

Approximately 65% of lower limb growth occurs close to the knee [12], with the distal femoral and proximal tibial growth plates contributing approximately 40% and 25%, respectively, of total lower limb growth [54]. Limb length discrepancy once growth has ceased is a major concern in children undergoing lower limb salvage surgery [2], with the compromise of the distal femoral physis being a significant factor in the outcome [45]. During the period of skeletal growth, one approach to minimizing the discrepancy in length is to reduce the growth of the contralateral limb through epiphysiodesis. When the estimated limb discrepancy is between 2 and 5 cm, it is recommended to halt the growth of the contralateral physis using epiphysiodesis. However, when the estimated difference in limb length is more than 5 cm, the recommendation is to perform secondary limb lengthening once skeletal growth ceases [55]. This procedure involves callus distraction using an extendable intramedullary nail or external fixation [54].

## Proximal femur

**Figure 7:**
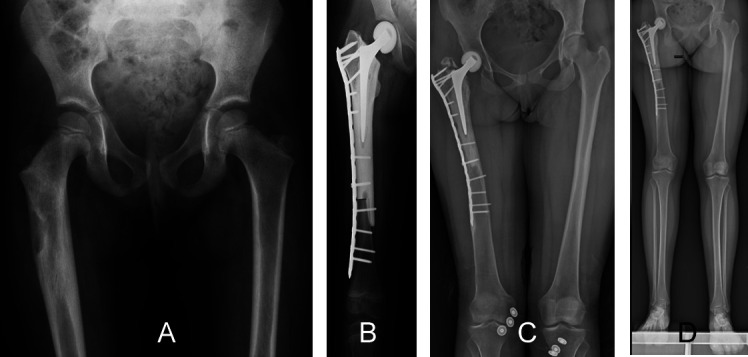
A 4-year-old girl diagnosed with Ewing sarcoma of the right proximal femur treated with osteoarticular resection and allograft–prosthesis composite reconstruction. (A) A preoperative radiograph showing an osteolytic lesion present in the subtrochanteric region of the proximal femur. (B) A postoperative radiograph shows reconstruction, using an allograft–prosthesis composite with a short stem and plate fixation together with reconstruction of the hip with a unipolar hemiarthroplasty with femoral ceramic head. Anteroposterior radiographic view (C) after 16 years of follow-up showing consolidation of the allograft, no loosening of the femoral prosthesis stem, growth of the remaining distal femur, and fracture of the greater trochanter of the allograft, and panoramic view (D) showing a discrepancy of 1.80 cm in limb length. Allograft–prosthesis composites may be an alternative to modular prosthesis in children with bone sarcoma of the proximal femur who have a small size of bone.

**Figure 8:**
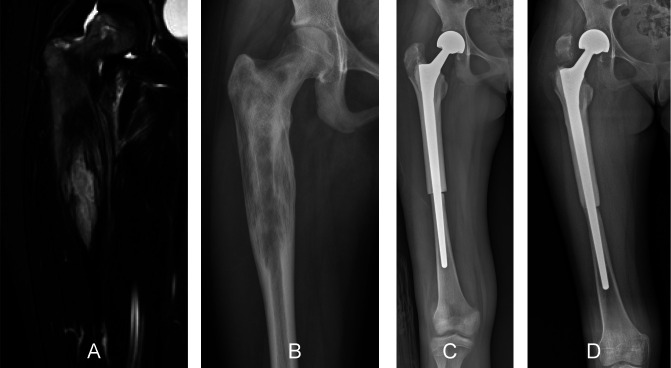
A 13-year-old girl, diagnosed with osteosarcoma, was treated with osteoarticular resection of the right proximal femur followed by reconstruction using an allograft–prosthesis composite. A coronal-T1 (A) magnetic resonance image shows a tumor extension to the proximal meta-diaphyseal region of the right femur. A preoperative anteroposterior radiographic view (B) showing an osteolytic lesion of the proximal femur. (C) Radiograph at one month postoperatively, showing the reconstruction using a press-fit femoral stem and reconstruction of the hip with bipolar hemiarthroplasty. (D) Radiograph at the 1-year follow-up showing fracture of the greater trochanteric region. Allograft–prosthesis composites may help preserve bone stock for future revision surgeries in children with bone sarcoma of the proximal femur.

Reconstruction options for children with bone sarcomas following proximal femur resection include a modular prosthesis or allograft–prosthesis composite (Figs. 7 and 8) [5, 9, 12]. One retrospective study analyzed the outcomes of 40 children who underwent primary bone tumor resection with subsequent reconstruction of the proximal femur [56]: 30 of the 40 children were treated with total arthroplasty [cemented (*n* = 27) or uncemented (*n* = 3) acetabular cup], while the remaining 10 were treated with hemiarthroplasty [56]. The risk of revision of the acetabular component in children over 11 years old at the time of surgery was 25%, while in children under 11, the risk was 75% [56]. All children with hemiarthroplasty suffered failure within nine years, due to pain or subluxation, and they underwent surgical revision with an uncemented acetabular cup [56]. In children treated with a cemented acetabular cup, the reconstruction survival was 62% at 10 years (*p* < 0.05) [56]. Although all children who were treated with an uncemented acetabular cup died from tumor-related disease, none required surgical revision of the reconstruction [56]. Complications related to the acetabular reconstruction may include instability and coxalgia, which in some children may lead to reoperation with total acetabular replacement, once skeletal maturity or triradiate cartilage fusion occurs [12, 56]. Manfrini et al. performed wide resection and reconstruction with an allograft and a free epiphyseal graft of the proximal fibula in a 4-year-old child with Ewing’s sarcoma of the proximal femur. The transplanted fibular head gradually enlarged, and 4 years after surgery, the hip joint was able to move at 100 degrees of flexion, 10 degrees of extension, and 30 degrees of abduction, and was able to walk with full weight bearing [57]. However, there have been no reports of similar reconstruction since then. Hip rotationplasty is an option for reconstruction after wide resection of proximal femoral sarcomas in very young children under 5 years of age in whom reconstruction with an expandable modular prosthesis or allograft-prosthesis composite is not possible. Eight patients underwent hip rotationplasty and all were able to perform full weight bearing and had good hip range of motion. Wound complications occurred in one patient. Hip rotationplasty not only provides better functional results than hip disarticulation, but the cartilage of the proximal tibia enters the acetabulum, allowing the development of a new femoral head [58]. Most of the studies analyzing proximal femur resections and reconstructions included both child and adult patients, leading to high heterogeneity among the population as reported in previous systematic reviews [59–61]. Therefore, there is a lack of data regarding reconstruction in children with bone sarcomas of the proximal femur [56].

## Proximal humerus

**Figure 9:**
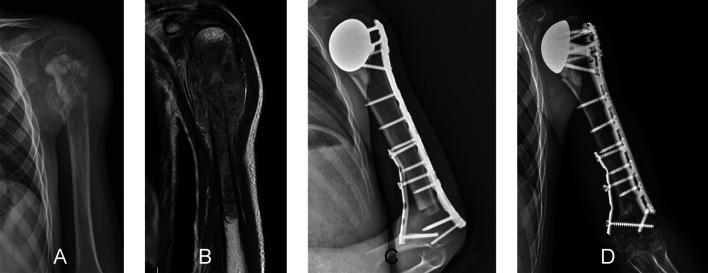
An 8-year-old boy with a diagnosis of osteosarcoma of the proximal humerus who was treated by osteoarticular resection and allograft–prosthesis composite reconstruction. A preoperative anteroposterior radiographic view (A) showing a lytic lesion of the proximal humerus. A coronal-T1 (B) magnetic resonance image showing the extent and location of the tumor. Radiographs showing allograft–prosthesis composite reconstruction at the 1-month (C) and 4-year (D) follow-up showing complete consolidation and integration of the allograft at the junction of the host bone and the graft. Allograft–prosthesis composites may be an alternative to modular prosthesis in children with bone sarcoma of the proximal humerus who have a small size of bone.

Unlike in the lower extremity, length discrepancy in children who underwent resection and reconstruction of the upper extremities for bone sarcoma is less of a functional problem and more of a cosmetic issue [2]. Reconstruction options for the proximal humerus of children following resection of bone sarcomas include biological reconstruction (such as clavicula pro humerus, free vascularized fibular graft, MBA or extracorporeal devitalized autograft) or prosthetic reconstruction (such as a modular prosthesis or allograft–prosthesis composite) (Fig. 9) [21], with prosthetic reconstruction being commonly used [21, 62]. A retrospective study of 25 children treated by prosthetic reconstruction of the proximal humerus reported complications in 27% of children, with infection being the most frequent complication. Subluxation was reported in nine of 25 children [21]. In another retrospective study of 35 children with proximal humerus bone sarcoma who underwent resection and reconstruction using an expandable prosthesis, the ten-year reconstruction survival rate was 75% [63]. Aseptic loosening was the most common cause of failure, occurring in 11% of children [63]. Subluxation was reported as the most common complication, occurring in 54% of children and the subluxation occurred in 86% of children under nine years old [63]. The authors suggested that, in these very young patients, biological reconstruction may be a better option [63]. An important limitation of prosthetic reconstructions after proximal humerus bone sarcoma resection in children is the small size of the humeral medullary canal and possible cortical atrophy of the bone around implanted stems [21]. Complications reported in very young children include aseptic loosening, cortical atrophy, or shoulder instability, which may need revision over time [62, 64].

An FVFG or clavicula pro humerus has been reported as a reconstruction option in very young children in which prosthetic reconstruction cannot be performed [63]. Epiphyseal grafts using a vascularized proximal fibula can be used to maintain the same length as it grows longitudinally. The average longitudinal growth of the graft has been reported to be approximately 5–17 mm per year [65–68]. The risk of complications following biological reconstructions ranges from 18% to 30%, and the risk of mechanical failure and avascular necrosis is very high [69–72]. One retrospective study analyzing 11 children who underwent reconstruction of the proximal humerus with an FVFG reported graft fracture in seven of 11 (64%) children, who required revision surgery [21]. Another retrospective study analyzing 11 children who underwent reconstruction with an FVFG following bone sarcoma resection in the proximal humerus, reported graft fracture in seven (64%) children, nerve palsy in four (36%), and avascular necrosis in two (18%) [73]. An FVFG has also been associated with a risk of fibular head resorption [72], and donor site morbidity [21, 71]. Clavicula pro humerus reconstruction may also carry a high risk of complications [70, 71, 74]. The two main complications are nonunion and limited shoulder motion [70, 71, 74]. This reconstruction option may be useful in very young children with involvement of the nondominant arm [75].

Regarding reconstruction of the proximal humerus using an allograft–prosthesis composite, previous studies have reported a 12–25% risk of revision [41, 76–78]. One retrospective study which included 11 patients treated using an allograft–prosthesis composite reconstruction following resection of the proximal humerus reported revision surgery in five of 11 (45%) patients. Instability was observed in four (36%), of whom three patients required reoperation for stabilization. Nonunion was observed in two of the 11 (18%) patients, and it was treated using bone grafting combined with osteosynthesis. Graft resorption occurred in one of the 11 (9%), who underwent revision with a strut allograft [77]. Another retrospective study analyzing 18 children treated with an allograft–prosthesis composite following resection of a bone tumor in the proximal humerus reported results in agreement with previous studies, suggesting that although the risk of complications following allograft–prosthesis composite reconstruction is high, it may be useful for bone stock preservation in very young children who may undergo future revision surgeries [10]. The competing risk analysis for reconstruction failure was 25% (95% CI [7% to 40%]). Four of 18 children underwent revision surgery for reconstruction failure: two children due to resorption of the proximal part of the allograft and two children due to graft fracture [10].

## Conclusions

The ideal method of intercalary reconstruction in children following resection of a bone tumor remains controversial [28]. While biological reconstructions seem to be the standard treatment following intercalary resection of bone sarcomas, there is still controversy about combining an MBA or tumor-devitalized autograft with an FVFG due to longer operative times and donor site morbidity [4]. Previous studies showed no difference in reconstruction survival when comparing children treated with an intercalary MBA and FVFG to those treated with an intercalary MBA alone [4, 32]. It appears that femoral intercalary allograft reconstruction carries a higher risk of subsequent fracture or nonunion compared to other long bone grafts [13, 34, 35]. FVFGs may improve bone healing in very long resections or may help rescue reconstructions of allograft or tumor-devitalized autograft alone following mechanical failures, such as fracture or nonunion [4, 7, 13, 20, 35–37]. Therefore, the use of an MBA with an FVFG seems to be recommended to reconstruct defects that are longer than 15 cm [4, 31, 34, 35]. However, the outcomes of intercalary reconstructions in most reported studies concerned only adult patients, with few reports describing their use in children [3, 4, 17, 30, 38, 79].

Reconstruction options following osteoarticular resection in children include a modular or expandable prosthesis or an allograft–prosthesis composite reconstruction. The risks of complications and implant survival seem to be comparable for these three surgical treatments. The ideal surgical option for osteoarticular reconstruction in children after bone tumor resection remains controversial. There is a lack of both quality and quantity of studies in the literature [9, 40, 55, 60, 62]. The risk of failure appears to be high following both biological and prosthetic reconstructions [9, 21, 40, 55, 60, 62]. However, while biological reconstructions or allograft–prosthesis composites have similar complications to prosthetic reconstructions, they may help preserve bone stock for future revision surgeries [10]. To select the best reconstruction option, it is necessary to take into consideration various factors including the age of the patient, bone size, availability of the chosen implant, technical expertise, and the preference of the surgeon.

**Figure 10:**
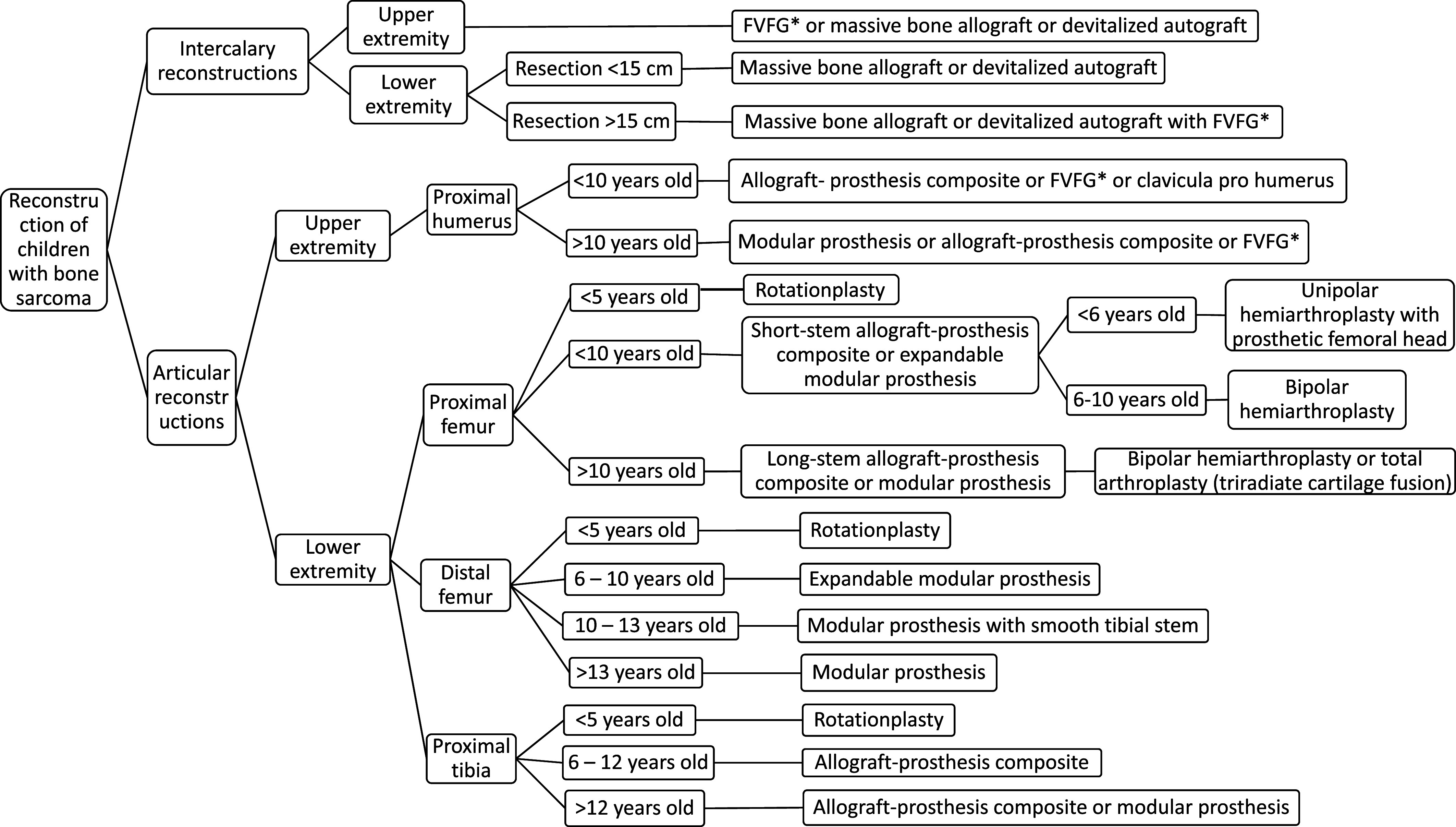
An algorithm for surgical treatment of children with bone sarcomas of the extremities. (*FVFG: free vascularized fibular graft).

Based on our review of the different reconstruction options in children with bone sarcomas, we propose an algorithm forsurgical treatments in children with bone sarcomas of the extremities (Fig. 10). There is limited data regarding reconstruction following intercalary or osteoarticular resection in children with bone sarcoma, and no studies comparing different reconstruction options. In addition, many articles reported pooled data that were obtained from patients of a wide range of ages and a range of anatomical sites. Therefore, there is a need for further research to confirm our algorithm and to help choose the optimal reconstruction approach in children with bone sarcomas of the extremities.

## Data Availability

The datasets generated, analyzed, or both during the present study are not publicly available because of privacy issues but are available from the corresponding author upon reasonable request.
